# Sociodemographic Factors Associated with Video Game Addiction in Schoolchildren from the South-Central Region of Chile

**DOI:** 10.3390/children11101156

**Published:** 2024-09-24

**Authors:** Alejandra Rodríguez-Fernández, Marcela Ruíz-De la Fuente, Eduard Maury-Sintjago, Sofía Petersen, Valentina Paredes, Bárbara Montero

**Affiliations:** 1 Department of Nutrition and Public Health, Universidad del Bío-Bío, Chillan 3780000, Chile; marcelaruiz@ubiobio.cl (M.R.-D.l.F.); emaury@ubiobio.cl (E.M.-S.); 2 Escuela de Nutrición y Dietética, Universidad del Bío-Bío, Chillan 3780000, Chile; sofia.petersen1901@alumnos.ubiobio.cl (S.P.); valentina.paredes1901@alumnos.ubiobio.cl (V.P.); barbara.montero1901@alumnos.ubiobio.cl (B.M.)

**Keywords:** schoolchildren, video games, addiction

## Abstract

Video game addiction is a worldwide concern, particularly in schoolchildren where it has impact on academic, social, and emotional spheres. The objective of this study was to determine the sociodemographic factors associated with video game addiction in schoolchildren from the south-central region of Chile. Methods: An analytical cross-sectional study that included a sample of 308 schoolchildren was performed. Sociodemographic factors were analyzed and levels of video game addiction was assessed using the Video Game Addiction Test (VAT) developed by Chóliz and Marco. A general linear regression model (ANCOVA) (α = 0.05) was applied (STATA v16). The prevalence of video game use was 82.7%, while addiction reached 10%. Risk factors associated with the increased risk of addiction were as follows: being male (β = 13.99; *p* < 0.001); being in the care of another relative (β = 11.1; *p* < 0.001); a higher number of people in the household (β = 11.2; *p* < 0.001); the caregiver in employment (β = 12.8; *p* < 0.001); and not performing extracurricular physical activity (β = 9.9; *p* < 0.001).

## 1. Introduction

After years of discussion about the relevance of recognizing video game dependency or addiction as a disorder, the eleventh revision of the International Classification of Diseases (ICD-11), published by the WHO in 2022, includes video gaming disorder, which refers to the addiction to digital gaming or video gaming, both online or offline [1].

The theories of video game addiction encompass various perspectives, such as the psychological dependence and structural factors of gaming. Shotton (1989) suggested that some users seek refuge in technology, while later studies, such as those by Ng and Wiemer-Hastings (2005), and Parke and Griffiths (2007), have addressed internet and game addiction. Rehbein et al. (2010) identified risk factors in adolescents, highlighting that 15-year-olds are more vulnerable [2,3,4,5]. Although King, Delfabbro, and Griffiths (2010b) emphasized the importance of rewards and punishments in video games over demographic factors [6], more recent research shows that gender, age, and family environment also play a role in addiction, being more common among adolescent boys [7].

Video game addiction is identified as a behavioral pattern characterized by persistent and recurrent gaming that involves the loss of control to stop playing, substantial time investment in gaming, underestimation of the impairment caused by the addictive behavior, and prioritization of the activity over any other activity [8,9]. In this sense, internet gaming disorder resembles pathological gambling and substance use disorders in key behavioral aspects related to withdrawal, tolerance, severe difficulty of reducing or quitting the behavior, and distress in daily life at the social, occupational, and family levels [10,11].

Reports have found that 97% of adolescents aged 12–17 play computer, web, portable, or console games in the United States [12,13]. An average of 48% of Europeans have played video games, and 87.4% of Norwegian young adults aged 16–50 play video games regularly, of whom 1.4% are considered addicted gamers and 7.3% problem gamers [14]. Among adolescents, the proportion of gamers is even higher, with 97% of Americans aged 12–17 playing video games [15]. In Latin America, Restrepo et al. showed that 26% of 12-year-old Colombian schoolchildren stated that they played video games every day [16]. Meanwhile in Chile, 8 out of 10 young people recognize their engagement in gaming, with an average of 13 h/week spent on online gaming [17].

Studies agree that males report more video-game-related problems compared to females, as do younger subjects [16,18,19]. In relation to the place of origin, Wittek et al. showed that the place of birth (Africa, Asia, and South and Central America) was positively associated with addicted and problem gamers [14].

Several studies have demonstrated that excessive engagement in video games can lead to negative consequences towards the academic, social, and emotional spheres [20,21]. Regarding academic achievement, poor academic performance and attention problems have been reported [22,23]. At the social level, addiction can lead to isolation and difficulties in establishing and maintaining healthy interpersonal relationships [24,25]. In terms of mental health, video-game-dependent youth may experience high levels of anxiety and depression [26,27]. Furthermore, prolonged exposure to video games can result in physical problems, such as a sedentary lifestyle and sleep disorders [28,29]. In Chile, this phenomenon has not been extensively studied, despite the growing penetration of technology and video games in the daily lives of young people [30,31].

The schooling context is a space that is critical for the development of children and adolescents, where not only academic knowledge but also social and emotional competencies are constructed [32]. Video game addiction can interfere with this process, affecting academic performance, interpersonal relationships, and the mental health of students [33,34]. In addition, sociodemographic factors such as socioeconomic status, family structure, and access to technology may have an influence on the prevalence and severity of this addiction [35,36]. Furthermore, it has been shown that the family environment plays a decisive role; under-involved parents or those who have poor parental monitoring skills may increase the likelihood of children developing a dependence on video games [37].

South-central Chile, which encompasses regions such as the Biobío, Ñuble and Araucanía, is characterized by particular sociodemographic and cultural aspects that may influence video game use and addiction among schoolchildren. These regions have experienced a growing urbanization process and steadily rising access to technological devices, which may be correlated with increased time devoted to video gaming [38].

In Chile, there have been no studies to date that analyze the risk factors associated with video game addiction in children, and the data are even scarcer in regions far from the capital. In this context, this study will address the most prevalent sociodemographic factors associated with video game addiction, the variability of this dependence as a function of sociodemographic characteristics, and how these factors are related to video game dependence in schoolchildren in the south-central region. The main objective is to identify and analyze these factors to provide relevant information and reduce the knowledge gap about this phenomenon in remote regions of the country.

## 2. Materials and Methods

### 2.1. Design

This is a quantitative, cross-sectional analytical study.

### 2.2. Sample and Inclusion Criteria

The initial sample consisted of 372 schoolchildren aged 14–15 years old who were willing to participate in the study and were enrolled in one of four public schools located in the south-central region of Chile. Of these individuals, 308 reported playing video games, and this number became the final sample size included in the analysis of this study. The sample calculation considered a significance level of 95%, an accuracy of 3%, and a prevalence or maximum variance of 50%.

The data collection procedure was conducted in three phases: (1) A study invitation email was sent to 10 schools located in the south-central region of Chile, where a total of 4 institutions agreed to participate, and participation was approved in meetings with school principals where information on the project and its objectives was provided; (2) Each school allowed researchers to contact the parents of the schoolchildren in parent meetings and in this way, to request authorization for minors to participate in the study by signing the informed consent form; (3) Schoolchildren who were authorized by their parents were asked to sign the informed consent form. Schoolchildren who did not sign the informed consent form were excluded. Data were gathered during the period from November 2022 to December 2023.

This research was previously reviewed and approved by the Bioethics Committee of the Universidad del Bío-Bío (Chile).

### 2.3. Study Variables

Sociodemographic characteristics: A survey was developed to record the following variables: sex (male/female), origin (urban/rural), caregiver (both parents, father only, mother only, other relatives), number of people in the household (1–2/3–5), whether the caregiver worked (yes/no), and whether the participant was engaged in extracurricular physical activity (EPA) (yes/no).

Video game addiction: The Video Game Addiction Test (VAT), developed by Chóliz and Marco, was used [10]. It consists of an instrument comprising 25 questions which uses an ordinal scale, where the value zero (0) is assigned to the answer “strongly disagree”, one (1) to “slightly disagree”, two (2) to “neutral”, three (3) to “slightly agree”, and four (4) to “strongly agree”. The full score is 100 points and the questionnaire is divided into four dimensions with their respective scores: (a) Withdrawal, which refers to emotional distress following the interruption or prolonged cessation of gaming (Items 3, 4, 6, 7, 10, 11, 13, 14, 21 and 25; total score 40 points), (b) Abuse and tolerance, where there is a progressive need to play more games or to play for longer periods of time (Items 1, 5, 8, 9 and 12; total score 20 points), (c) Problems caused by video games, which refers to spending excessive time on video-game-related activities, even interfering with the activities of daily living (Items 16, 17, 19 and 23; total score 16 points), (d) Control difficulties, which refers to difficulties in stopping play, despite the fact that it was not appropriate or functional to do so at that time or situation (Items 2, 15, 18, 20, 22 and 24; total score 24 points). The higher the score, the greater the addiction. Since the instrument did not have a cut-off score, the prevalence of video game addiction was estimated using the total scores above the 75th percentile. The internal consistency of the instrument was evaluated, obtaining a Cronbach’s alpha value of 0.9, in agreement with values reported by Luján-Cabrera et al. (2023) for the Ibero-American population [39].

### 2.4. Statistical Analysis

Univariate, bivariate, and multivariate descriptive data analyses were conducted. Numerical variables were described with the mean and standard deviation while categorical variables were described with the absolute frequency and percentage. The Student’s *t* test was used to assess the bivariate relationships between independent samples. Finally, a general linear regression model (ANCOVA) was applied to assess the association between predictors and the average score of the Video Game Addiction Test and its dimensions. Regression equations were estimated for each dimension and for the total scale according to risk factors, which were grouped together in the final model obtained using the stepwise method. Data processing was performed using the STATA v16 statistical software, considering a significance level of α < 0.05.

## 3. Results

The prevalence of video game use was 82.7%, while dependence on video games reached 10%. In relation to the sociodemographic characteristics of the 308 schoolchildren, males accounted for 51% and were mainly from urban areas (67.5%). Most of the children were in the care of one or both parents (53.3%), while 46.7% were cared for by another relative. The number of people in the household corresponded mostly to 1–2 members (54.6%), the caregiver was employed in 85.7% of the cases, and 68.8% of the minors were not engaged in extracurricular physical activity (Table 1).

The results of the Video Game Addiction Test (VAT) indicated that in the abstinence dimension, participants scored an average of 14.9 out of 40 points, while in abuse and tolerance, the average was 9.1 out of 20. In terms of problems caused by video games, the average was 6.9 out of 14 (Table 2).

When evaluating the association between video game addiction and sociodemographic characteristics, significant differences were found for sex in all dimensions and in the overall questionnaire score, with dependence being higher in males (*p* < 0.001). The highest score according to origin was obtained by urban residents in all dimensions (*p* < 0.001). Regarding the caregiver, the score was significantly higher in all dimensions for minors who were in the care of another relative (*p* < 0.001). In addition, living in a household with more than three people increased the average score in all dimensions when compared to households with 1–2 members (*p* < 0.001). The highest values were obtained by individuals whose caregiver had a job (*p* < 0.001). Finally, extracurricular physical activity was associated with lower scores (*p* < 0.001). (Table 3).

The multiple regression model for each dimension of the VAT along with sociodemographic characteristics showed that in the dimension of withdrawal, there was an association with sex, as being male was identified as a risk factor and increased the score by six points when compared to females (β = 6.0; *p* < 0.001). Being in the care of a relative increased the risk of dependence by 3.6 points compared to minors who were cared for by one or both of their parents (β = 3.58; *p* < 0.001). In addition, living in a household with more than three people increased the risk of withdrawal by nearly six points (β = 5.73; *p* < 0.001), as well as when the person responsible for the student had a job, in which case increased the value by almost eight points (β = 7.67; *p* < 0.001). Moreover, not performing extracurricular physical activity increased withdrawal by four points when compared to individuals who did (β = 4.11; *p* < 0.001). Regarding the dimension of abuse and tolerance, the risk factors were the same as those for the withdrawal dimension, where being male increased the risk by 2.51 points, being cared for by relatives increased the risk by 3.4 points, and living in a household with more than three people increased the risk by almost 2 points. If the caregiver had a job, this dimension increased by more than 2 points, and not performing extracurricular physical activity increased the score by an average of 1.6 points. For the dimension of problems caused by video games, risk factors were as follows: being male (β = 1.44; *p* < 0.001), being cared for by a relative (β = 1.2; *p* < 0.001), the person responsible for the student had a job (β = 2.16; *p* < 0.001), and not performing extracurricular physical activity (β = 1.6; *p* < 0.001). In this dimension, being of rural origin acted as a protective factor, decreasing the score (β = −2.2; *p* < 0.001). For the dimension of control difficulties, risk factors were being male, being cared for by a relative, living in a household with more than three people, and not performing extracurricular physical activity. Being of rural origin decreased the risk for this dimension by almost four points (β = −3.76; *p* < 0.001). Finally, when assessing the total score, all of the predictor variables were associated with video game addiction since they were constituted as risk factors, with the exception of rural origin, which acted as a protective factor (Table 4).

## 4. Discussion

The increasing accessibility to video games is steadily rising in the public interest. Moreover, video games have become a part of adolescents’ everyday lives. Although a positive impact has been shown in fields such as entertainment and education [40], it has also been found that the excessive use of video games poses risks to players, such as the negative influences on their lives, sleep, academic performance, and social relationships [24,25,26,27,31,41].

In our sample of adolescents, the prevalence of video game use was 82.7%, similar to that reported in another Chilean study [42]. Furthermore, video game addiction or excessive and dysfunctional use among gamers [43] has a worldwide prevalence of up to 5.8% [44,45]. It is 2.5 times higher in males than in females [46], and also more addictive [47,48]. In this study, the prevalence of dependence was 10%, with male adolescents showing higher problematic use in the four dimensions examined: withdrawal; abuse and tolerance; problems caused by video games; and control difficulties (*p* < 0.001). In addition, the regression analysis found that being male increased the risk of video game addiction by 13.9 points. This was the greatest determining factor among those analyzed, which was similar to findings reported in other studies [49,50]. These findings are also in line with those found in another Chilean study, where a high prevalence (10–15%) of dependence on video games was reported in schoolchildren aged 9–16 years old, with similar and more addictive behaviors observed in male subjects [51]. This may be due to biological and/or social aspects, for example, Wang et al. (2019) reported a sexual dimorphism of the brain, where males tend to have increased cortical thickness in the key areas of executive control, such as the middle frontal gyrus and superior parietal lobe, which are linked to cognitive control, visual attention, and impulsivity [52]. On the other hand, from a social point of view, it is important to consider several aspects. Historically, video games have been co-marketed more towards the male audience through representations and themes that are considered more attractive to men, such as action, war, and sports [53]. However, the representation of female characters has been limited or hypersexualized, which can discourage women to identify with video games [54].

In the analyzed group, participants from urban areas showed higher problematic use and dependence on video games compared to schoolchildren from rural areas, similar to what has been reported [41]. Similarly, living in an urban area increased the risk of addiction by 8.56 points, which could be related to internet access and connectivity [36].

A higher number of people in the household was associated with a higher dependence in two of the four dimensions analyzed: withdrawal, and control difficulties (*p* < 0.001). In addition, if the caregiver responsible for the adolescent was in employment, this resulted in a higher dependence in three of the four dimensions: withdrawal, abuse and tolerance, and problems caused by video games (*p* < 0.001). Moreover, the risk factor for dependence increased by 12.8 points, and became the second-most relevant variable among the socioeconomic variables analyzed. Similarly, a study conducted in South Korea found that parents who worked long hours constituted a risk factor for the development of addiction, since their children lacked the ability to regulate self-control [55].

When the adolescent was in the care of another relative, higher problematic dependent use was observed in the four dimensions (<0.001); additionally, this situation increased the risk of dependence by 11.7 points. It has become increasingly common for relatives, usually grandparents, to take care of adolescents, thus contributing to the stabilization of the family’s employment situation and economic income [56]. In addition, children raised by their grandparents are more likely to lose respect for authority, have looser boundaries and rules, and experience role confusion [57], and if this setting is related to low income, there may be a higher likelihood for adolescents to become addicted to the internet and gaming [51,58,59]. Conversely, a close parent-adolescent relationship is a protective factor against problematic screen use [7].

Adolescents who reported a lack of practicing extracurricular physical activity also showed a higher dependence on video games in all the dimensions studied (*p* < 0.001), and increasing the risk of dependence by almost 10 points. A higher dependence on video games facilitates sedentary behavior in adolescents, which, along with the indirect effect of video games on the onset of sleep disorders [30,31] and inadequate eating habits, increases the probability of suffering health problems and malnutrition due to excessive play [28]. It is beyond disputed that video game addiction has a greater impact on a vulnerable age group such as adolescents, and therefore, risk factors and consequences related to this situation are worthy of further investigation.

Given the prevalence and health consequences of this type of dependence, this issue must be addressed as a public health problem similar to other addictive behaviors (e.g., alcoholism, tobacco use) through promotion, prevention, and treatment strategies. Considering the critical role that parents play in the upbringing and habits of their children, it is imperative that they are instructed in this subject. Parents should know about the negative aspects of the indiscriminate use of video games and appreciate the importance in developing a close relationship and having open communication with their children in order to protect them from the problematic usage of video games [37,51].

This study was not free of limitations, such as the number of participants, and did not consider other variables such as the amount of video gaming hours, reasons to play video games, the convenience sample, self-reporting, and social desirability among others. It should also be noted that since this is a cross-sectional study, it was not possible to interpret causality.

## 5. Conclusions

The findings of this study reveal that the use of video games is highly prevalent among schoolchildren, with a significant rate of dependence associated with factors such as being male, residing in urban areas, being cared for by family members other than parents, living in households with a larger number of members, and not practicing extracurricular physical activities. These results underscore the importance of addressing video game dependence as a public health problem, similar to other addictions, due to its negative impact on academic performance, social relationships, and the general health of adolescents. Despite the limitations of the study, such as the nature of the sampling and cross-sectional study, these findings offer a starting point for future research on educational and preventive interventions that can reduce video game dependence. In addition, they contribute to educational practice by highlighting the need to involve parents and teachers in the regulation of video game use and the promotion of healthier habits, such as physical activity and effective communication in the family environment.

## Figures and Tables

**Table 1:** The sociodemographic characteristics of the schoolchildren.

Sociodemographic Characteristics and VAT Score by Dimensions	n = 308	%
Sex		
Female	152	49.4
Male	156	50.7
Origin		
Urban	208	67.5
Rural	100	32.5
Caregiver		
Both parents/father/mother	164	53.3
Other relatives	144	46.7
Number of people in the household		
1–2	168	54.6
3–5	140	45.5
Does your caregiver have a job?		
Yes	264	85.7
No	44	14.3
Do you perform extracurricular physical activity?		
No	196	68.8
Yes	112	31.2

**Table 2:** The average score on the Video Game Addiction Test (VAT).

Video Game Addiction Test	$\bar{x}$ ± SD	Min	Max	Median
Withdrawal (40 points)	14.9 ± 9.5	0	35	12
Abuse and tolerance (20 points)	9.1 ± 5.1	0	20	9
Problems caused by video games (14 points)	6.9 ± 3.7	0	14	6
Control difficulties (21 points)	8.9 ± 6.4	0	21	7
Total (100 points)	39.9 ± 20.9	9	75	33

**Table 3:** Relationship between video game addiction and sociodemographic characteristics.

Sociodemographic Characteristics	Withdrawal	Abuse and Tolerance	Problems Caused by Video Games	Control Difficulties	Total Video Games
	$\bar{x}$ ± SD	$\bar{x}$ ± SD	$\bar{x}$ ± SD	$\bar{x}$ ± SD	$\bar{x}$ ± SD
Sex					
Female	11.3 ± 7.7	7.7 ± 5.1	5.8 ± 3.5	5.9 ± 3.9	30.4 ± 15.2
Male	18.7 ± 9.3	10.6 ± 4.5	7.9 ± 3.6	11.8 ± 5.1	49.2 ± 18.5
*p*	<0.001	<0.001	<0.001	<0.001	<0.001
Cohen’s Δ	0.87	0.61	0.56	1.31	1.11
Origin					
Urban	16.6 ± 9.6	9.8 ± 4.9	7.8 ± 3.8	10.8 ± 5.7	45.1 ± 22.1
Rural	11.4 ± 7.5	7.7 ± 5.1	4.9 ± 2.9	5.1 ± 3.0	31.6 ± 12.4
*p*	<0.001	0.0004	<0.001	<0.001	<0.001
Cohen’s Δ	0.61	0.42	0.87	1.31	0.78
Caregiver					
Both parents/father/mother	12.4 ± 8.1	7.1 ± 3.8	5.9 ± 3.1	6.9 ± 4.2	32.2 ± 18.2
Other relatives	17.2 ± 7.8	10.9 ± 4.5	7.8 ± 3.5	10.5 ± 5.8	46.5 ± 19.4
*p*	<0.001	<0.001	<0.001	<0.001	<0.001
Cohen’s Δ	0.60	0.92	0.58	0.72	0.76
Number of people in the household					
1–2	12.2 ± 7.1	8.3 ± 3.8	6.5 ± 3.4	6.7 ± 44	33.7 ± 16.5
3–5	18.2 ± 6.8	10.2 ± 5.1	7.4 ± 3.2	11.5 ± 6.2	47.2 ± 20.6
*p*	<0.001	0.0009	0.009	<0.001	<0.001
Cohen’s Δ	0.86	0.43	0.27	0.91	0.73
Does your caregiver have a job?					
Yes	14.3 ± 5.8	8.6 ± 3.48	6.4 ± 3.6	8.5 ± 6.4	37.3 ± 19.8
No	22.4 ± 6.9	11.8 ± 41	9.6 ± 3.2	11.2 ± 5.8	55.4 ± 17.2
*p*	<0.001	0.0001	<0.001	0.0085	<0.001
Cohen’s Δ	1.27	0.84	0.94	0.44	0.98
Do you perform extracurricular physical activity?					
No	13.1 ± 7.6	8.1 ± 4.3	6.2 ± 3.1	7.4 ± 5.3	34.8 ± 18.6
Yes	18.3 ± 9.5	10.8 ± 5.4	8.2 ± 3.7	11.5 ± 6.6	48.7 ± 20.2
*p*	<0.001	<0.001	<0.001	<0.001	<0.001
Cohen’s Δ	0.61	0.56	0.59	0.69	0.72

The Student’s *t* test was used for independent samples.

**Table 4:** The results from using the general linear model (ANCOVA) to estimate risk factors in the VAT for all dimensions and the overall total score.

Sociodemographic Characteristics	Withdrawal		Abuse and Tolerance		Problems Caused by Video Games		Control Difficulties		Total	
	b	*p*	b	*p*	b	*p*	b	*p*	b	*p*
Full Model (Adjusted for age)										
Sex: Male	5.6	<0.001	2.31	<0.001	1.29	0.001	4.74	<0.001	13.99	<0.001
Origin: Rural	−1.82	0.049	−0.89	0.097	−2.13	<0.001	−3.71	<0.001	−8.56	<0.001
Caregiver: Another relative	3.6	<0.001	3.39	<0.001	1.27	0.001	2.87	<0.001	11.1	<0.001
Number of people in the household: 3–5	5.6	<0.001	1.8	<0.001	0.695	0.066	3.58	<0.001	11.2	<0.001
Caregiver: works	7.5	<0.001	2.12	0.004	2.34	<0.001	0.82	0.291	12.8	<0.001
Extracurricular PA: No	3.9	<0.001	1.5	0.006	1.48	<0.001	2.96	<0.001	9.9	<0.001
Stepwise Model (Adjusted for age)										
Sex: Male	6.0	<0.001	2.51	<0.001	1.44	0.001	4.90	<0.001		
Origin: Rural					−2.20	0.001	−3.76	<0.001		
Caregiver: Another relative	3.58	<0.001	3.38	<0.001	1.20	0.002	2.91	<0.001		
Number of people in the household: 3–5	5.73	<0.001	1.89	<0.001			3.47	<0.001		
Caregiver: works Yes	7.67	<0.001	2.22	0.003	2.16	<0.001				
Extracurricular PA: No	4.11	<0.001	1.60	0.004	1.57	<0.001	2.97	<0.001		
Final Model Equation										
Withdrawal	Withdrawal = 4.77 + 6.0_(Male)_ + 3.58_(AnotherRelative)_ + 5.73_(3–5peopleinthehousehold)_ + 7.5_(caregiverworks)_ + 4.11_(DoesNotPerformPA)_									
Tolerance	AbuseandTolerance = 4.30 + 2.50_(Male)_ + 3.38_(AnotherRelative)_ + 1.89_(3–5peopleinthehousehold)_ + 2.21_(caregiverworks)_ + 1.59_(DoesNotPerformPA)_									
Problems	Problems =5.35 + 1.44_(Male)_ − 2.20_(Rural)_ + 1.2_(AnotherRelative)_ + 2.16_(caregiverworks)_ + 1.58_(DoesNotPerformPA)_									
Control	ControlDifficulties = 3.43 + 4.90_(Male)_ − 3.76_(Rural)_ + 2.91_(AnotherRelative)_ + 3.46_(3–5peopleinthehousehold)_ + 2.98_(DoesNotPerformPA)_									
Total	Total = 18.8 + 13.9_(Male)_ − 8.56Rural + 11.1_(AnotherRelative)_ + 11.7_(3–5peopleinthehousehold)_ + 12.8_(caregiverworks)_ + 9.94_(DoesNotPerformPA)_									

## Data Availability

The data presented in this study are available on request from the corresponding author.
